# LncRNA MIR205HG accelerates cell proliferation, migration and invasion in hepatoblastoma through the activation of MAPK signaling pathway and PI3K/AKT signaling pathway

**DOI:** 10.1186/s13062-021-00309-3

**Published:** 2022-01-07

**Authors:** Wei Zhang, Feng Liang, Qingfeng Li, Hong Sun, Fei Li, Zhibo Jiao, Jie Lei

**Affiliations:** 1 Department of Pediatric Surgery, the First Affiliated Hospital of Hebei North University, No. 12 Changqing Road, Qiaoxi District, Zhangjiakou, 075000 Hebei China; 2 Department of Hepatobiliary Surgery, The First Affiliated Hospital of Hebei North University, Zhangjiakou, 075000 Hebei China

**Keywords:** Hepatoblastoma, MIR205HG, miR-514a-5p, miR-205-5p, MAPK9

## Abstract

**Background:**

Hepatoblastoma (HB) is identified to be the most common liver malignancy which occurs in children. Long non-coding RNAs (lncRNAs) have been implicated in numerous biological processes and diseases, including HB. LncRNA MIR205 host gene (MIR205HG) has been investigated in multiple cancers, however, its role in HB remains to be elucidated.

**Methods:**

MIR205HG expression was analyzed by RT-qPCR. EdU, colony formation and transwell assays were implemented to measure the biological function of MIR205HG on the progression of HB. Mechanism assays were carried out to probe into the underlying mechanism of MIR205HG in HB cells.

**Results:**

MIR205HG was significantly overexpressed in HB. Moreover, MIR205HG inhibition suppressed the proliferative, migratory and invasive capacities of HB cells. Furthermore, MIR205HG competitively bound to microRNA-514a-5p (miR-514a-5p) and targeted mitogen-activated protein kinase 9 (MAPK9) to stimulate mitogen activated protein kinase (MAPK) signaling pathway. Besides, MIR205HG also served as a sponge for microRNA-205-5p (miR-205-5p) to activate the PI3K/AKT signaling pathway.

**Conclusion:**

MIR205HG drives the progression of HB which might provide an efficient marker and new therapeutic target for HB.

**Supplementary Information:**

The online version contains supplementary material available at 10.1186/s13062-021-00309-3.

## Background

HB is the most common primary liver tumor which frequently occurs in infants and in children younger than 5 years [1]. It accounts for more than 25% pediatric hepatic tumors and almost half of those are malignant [2]. It has been reported that the occurrence of HB is correlated with Beckwith-Weidemann syndrome, familial adenomatosis polypi as well as low birth weight [3]. For the treatment of HB, complete surgical removal is of great significance in curing HB. However, the tumor is unresectable due to its extensive hepatic involvement [4], leading to the unsatisfactory overall survival rate and poor prognosis of HB patients [5, 6]. Thence, exploring therapeutic strategies of HB from a novel insight may be helpful for the treatment of HB.

Long non-coding RNAs (lncRNAs) have been identified as valuable therapeutic targets for cancers due to their distinctive roles in cancers [7]. For instance, Li et al. have demonstrated that TUG1 exacerbates the initiation of epithelial ovarian cancer via enhancing AURKA expression (8). Luo et al. have discovered that DANCR plays a promoting role in pancreatic cancer through serving as a sponge for miR-33b [9]. Yan et al. have revealed that LINC00052 plays the suppressive role in hepatocellular carcinoma [10]. The significance of lncRNAs has also been implicated in HB. In addition, Zhang et al. have validated that OIP5-AS1 boosts the development of HB via targeting miR-186a-5p/ZEB1 axis [11]. Chen et al. have elucidated that lncRNA CRNDE influences the angiogenesis of HB cells through functioning as a competing endogenous RNA (ceRNA) [12]. Cui et al. have substantiated that lncRNA ZFAS1 accelerates tumor growth in HB by sequestering miR-193a-3p [13].

As a common lncRNA, MIR205HG has been also investigated in multiple malignancies, including esophageal cell carcinoma, lung squamous cell carcinoma, cervical cancer and so on [14–16]. In this study, we probed into the effects of MIR205HG on the progression of HB and investigated into the latent regulatory mechanism.

## Results

### LncRNA MIR205HG is overexpressed in HB and mainly located in cytoplasm of HB cells

**Fig. 1:**
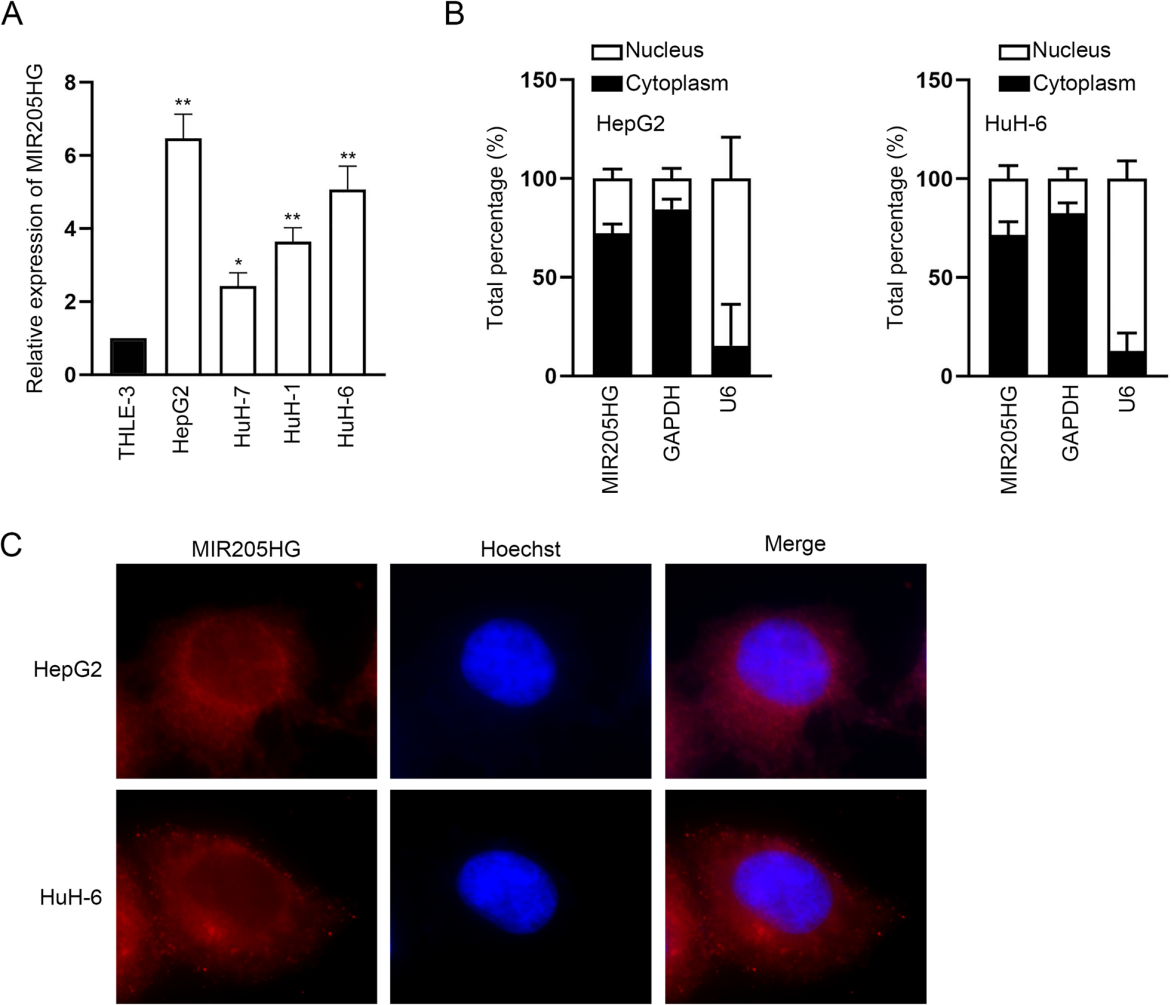
LncRNA MIR205HG is overexpressed in HB and mainly located in cytoplasm of HB cells. **A** RT-qPCR detected MIR205HG expression in HB cell lines and normal cell line. **B**–**C** Subcellular fractionation and FISH assays analyzed the subcellular distribution of MIR205HG in HB cells. GAPDH and U6 were used as internal controls for RT-qPCR. Each experiment was performed in triplicate. Student’s *t* test and one-way ANOVA were adopted for statistics. *P < 0.05, **P < 0.01

According to GEO database (https://medworm.com/journal/geo-gene-expression-omnibus/), we discovered that lncRNA MIR205HG was up-regulated in HB tissues. Therefore, MIR205HG was chosen for our investigation. Firstly, RT-qPCR was used to examine MIR205HG expression in HepG2, HuH-7, HuH-1, HuH-6 and THLE-3 cell lines, and the results suggested that MIR205HG expression was distinctly high in HB cell lines (HepG2, HuH-7, HuH-1, HuH-6) compared to normal cell line (THLE-3) (Fig. 1A). Meanwhile, the subcellular location of MIR205HG in HB cells was explored through subcellular fractionation and FISH assays. The experimental results manifested that MIR205HG was prominently distributed in the cytoplasm of HepG2 and HuH-6 cells (Fig. 1B–C). To be summarized, MIR205HG is overexpressed in HB and mainly located in cytoplasm of HB cells.

### MIR205HG contributes to cell proliferation, migration and invasion in HB cells

**Fig. 2:**
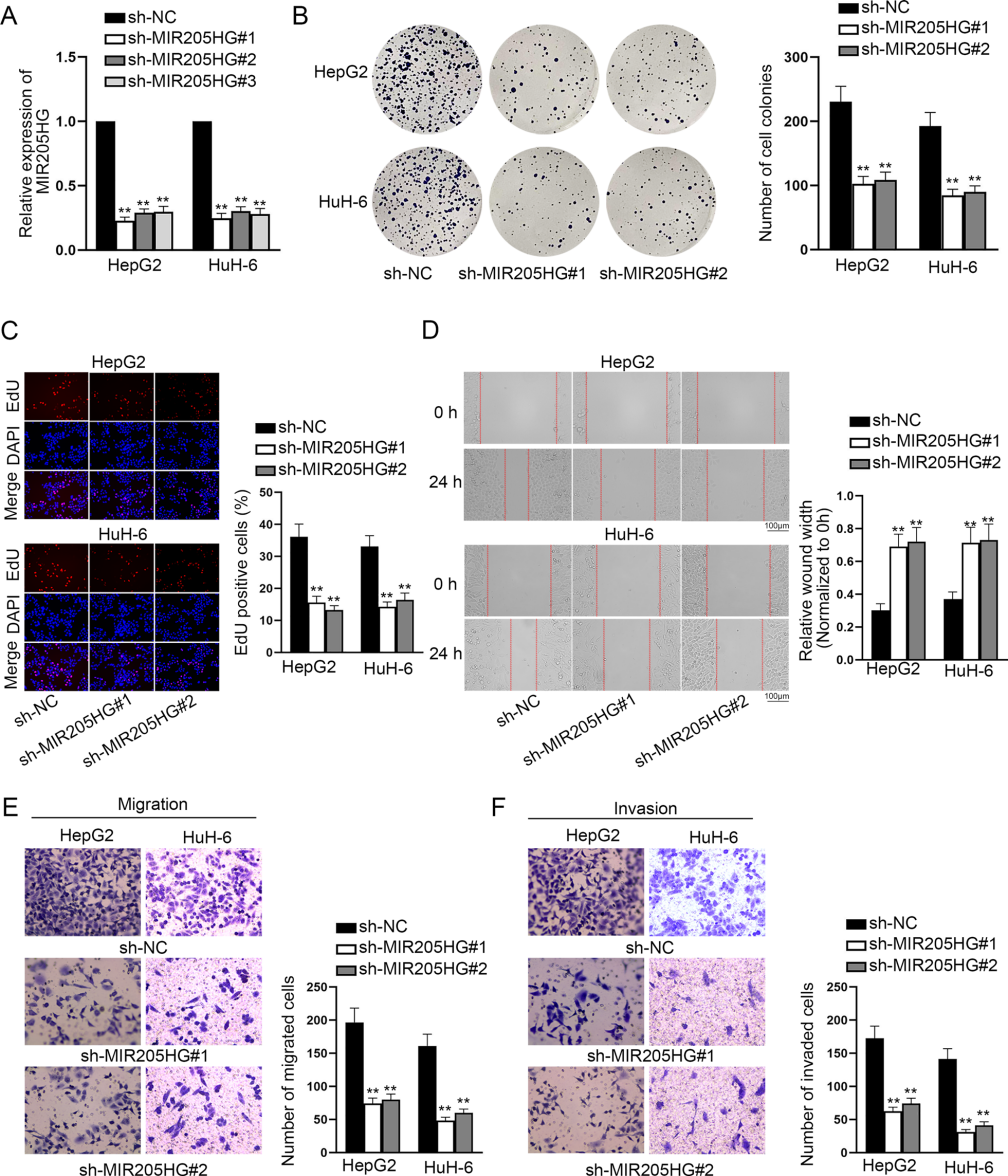
MIR205HG contributes to cell proliferation, migration and invasion in HB cells. **A** MIR205HG expression was cut down in HepG2 and HuH-6 cells. **B**–**C** Colony formation and EdU assays evaluated the proliferative capacity of HB cells after MIR205HG was silenced. **D** The migratory capacity of HB cells was evaluated by wound healing assay. **E**–**F** Transwell assays were implemented to observe the migration and invasion of HB cells. Each experiment was performed in triplicate. One-way ANOVA was used for statistics. **P < 0.01

To evaluate the function of MIR205HG on HB progression via loss-of-function assays, MIR205HG expression was firstly cut down in HepG2 and HuH-6 cells (Fig. 2A). Due to the relative higher efficiency, sh-MIR205HG#1 and sh-MIR205HG#2 were chosen for the follow-up assays. We conducted colony formation and EdU assays to detect the effect of MIR205HG on HB cell proliferation. It’s unmasked by the results that the proliferation of HepG2 and HuH-6 cells was impeded when MIR205HG was down-regulated (Fig. 2B–C). In addition, the experimental results of wound healing and transwell assays disclosed that the migratory ability of HB cells was accordingly attenuated after MIR205HG was silenced (Fig. 2D–E). Subsequently, transwell assay using Matrigel was conducted to examine cell invasion in HepG2 and HuH-6 cells. It’s shown by the results that the number of invaded cells was also decreased when MIR205HG was interfered (Fig. 2F). For further verification, we performed gain-of-function assays. MIR205HG expression was increased by pcDNA3.1/MIR205HG in THLE-3 cells (Additional file 2: Fig. S1A). The results of EdU assay demonstrated that, the proliferation of THLE-3 cells was enhanced when MIR205HG was up-regulated (Additional file 2: Fig. S1B). Moreover, the results of transwell assay showcased that the migratory and invasive capacities of THLE-3 cells was enhanced after the overexpression of MIR205HG (Additional file 2: Fig. S1C–D). Moreover, apart from the in vitro experiments, we performed animal experiments to detect the function of MIR205HG in vivo. As indicated in Additional file 2: Fig. 1E–F, we discovered that MIR205HG ablation decreased the volume and weight of tumor. Taken together, MIR205HG plays a promoting role in the development of HB.

### MIR205HG binds to miR-514a-5p in HB cells

**Fig. 3:**
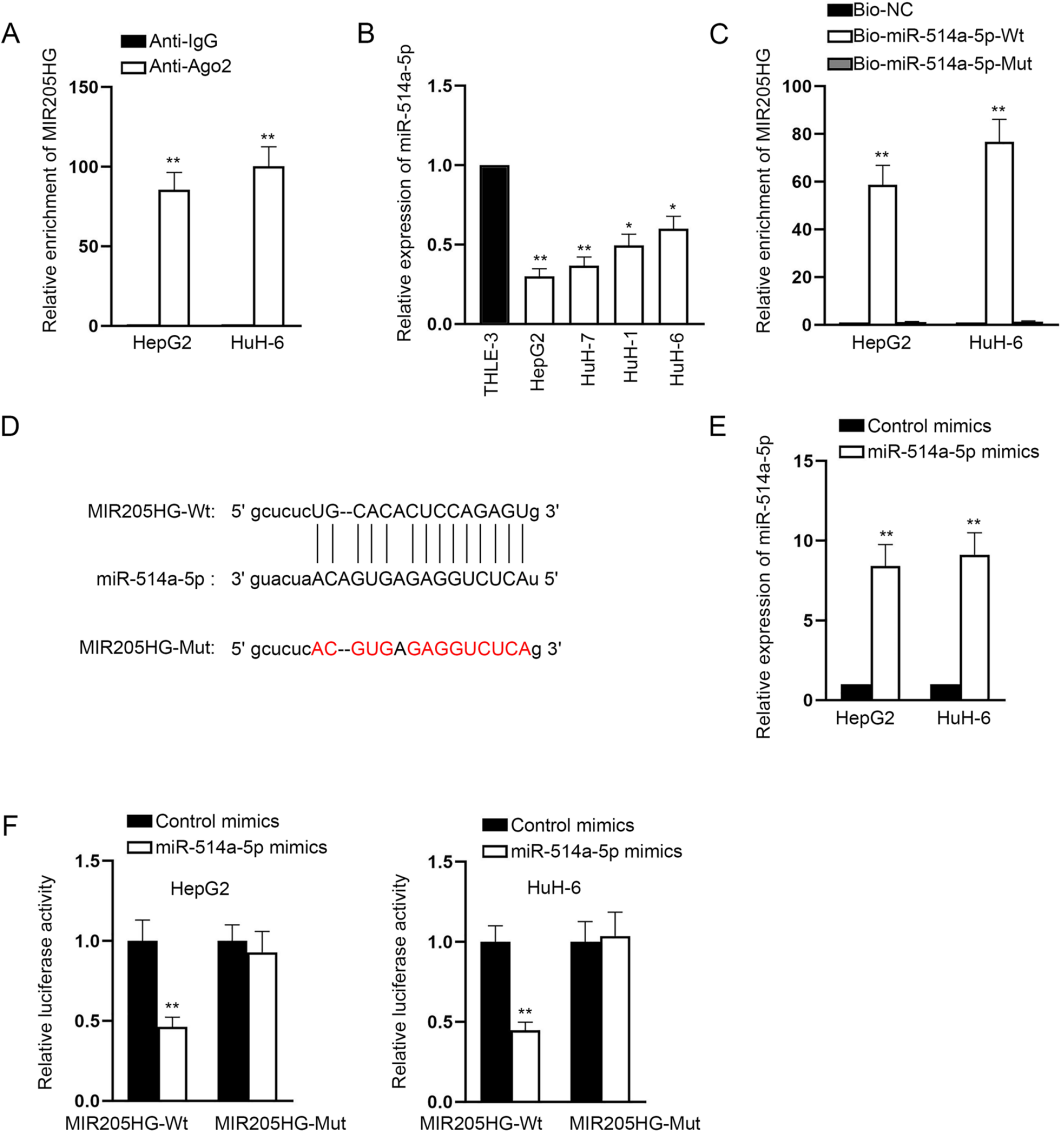
MIR205HG binds to miR-514a-5p in HB cells. **A** RIP assay exhibited the affinity of MIR205HG with Ago2 protein. **B** MiR-514a-5p expression in HB cells was examined by RT-qPCR. **C** RNA pull down assay verified the relationship between MIR205HG and miR-514a-5p. **D** Binding regions between MIR205HG and miR-514a-5p. **E** MiR-514a-5p expression was enhanced by transfection of miR-514a-5p mimics. **F** Luciferase reporter assay examined the luciferase activity in MIR205HG-Wt group and MIR205HG-Mut group after transfection of miR-514a-5p mimics. GAPDH and U6 were used as internal references for RT-qPCR. Each experiment was performed in triplicate. Student’s *t* test, one-way ANOVA and two-way ANOVA were adopted for statistics. *P < 0.05, **P < 0.01

Based on the previous findings of subcellular fractionation and FISH assays, we made a hypothesis that MIR205HG might act as a ceRNA at post-transcriptional level. For validation, RIP assay attested that MIR205HG was enriched in precipitates by Ago2 antibodies (Fig. 3A), which indicated the existence of MIR205HG in RNA-induced silencing complex (RISC). This indicated that MIR205HG may be a ceRNA as RISC is the major workplace for miRNAs [17]. With the application of starBase (http://starbase.sysu.edu.cn/index.php), it’s discovered that only miR-514a-5p was overtly down-regulated in HB cells among the predicted 12 miRNAs (Supplementary Table 1). RT-qPCR analysis was performed to detect miR-514a-5p expression in HB cells and normal cells, uncovering that miR-514a-5p was notably down-regulated in HB cells (Fig. 3B). Subsequently, we performed RNA pull down assay to detect the binding of MIR205HG with miR-514a-5p. The results showed that MIR205HG was enriched in Bio-miR-514a-5p-Wt instead of Bio-miR-514a-5p-Mut, indicating the interaction between them (Fig. 3C). As exhibited in Fig. 3D, the binding regions between MIR205HG and miR-514a-5p were acquired based on the data from starBase. To elevate the expression of miR-514a-5p, miR-514a-5p mimics were transfected into HepG2 and HuH-6 cells (Fig. 3E). Afterwards, we performed luciferase reporter assay to further prove the interaction between MIR205HG and miR-514a-5p. It’s unmasked that when miR-514a-5p was overexpressed, the luciferase activity of MIR205HG-Wt was reduced (Fig. 3F). In conclusion, miR-514a-5p binds to MIR205HG.

### MIR205HG modulates MAPK9 expression and activates MAPK signaling pathway by competitively binding to miR-514a-5p

**Fig. 4:**
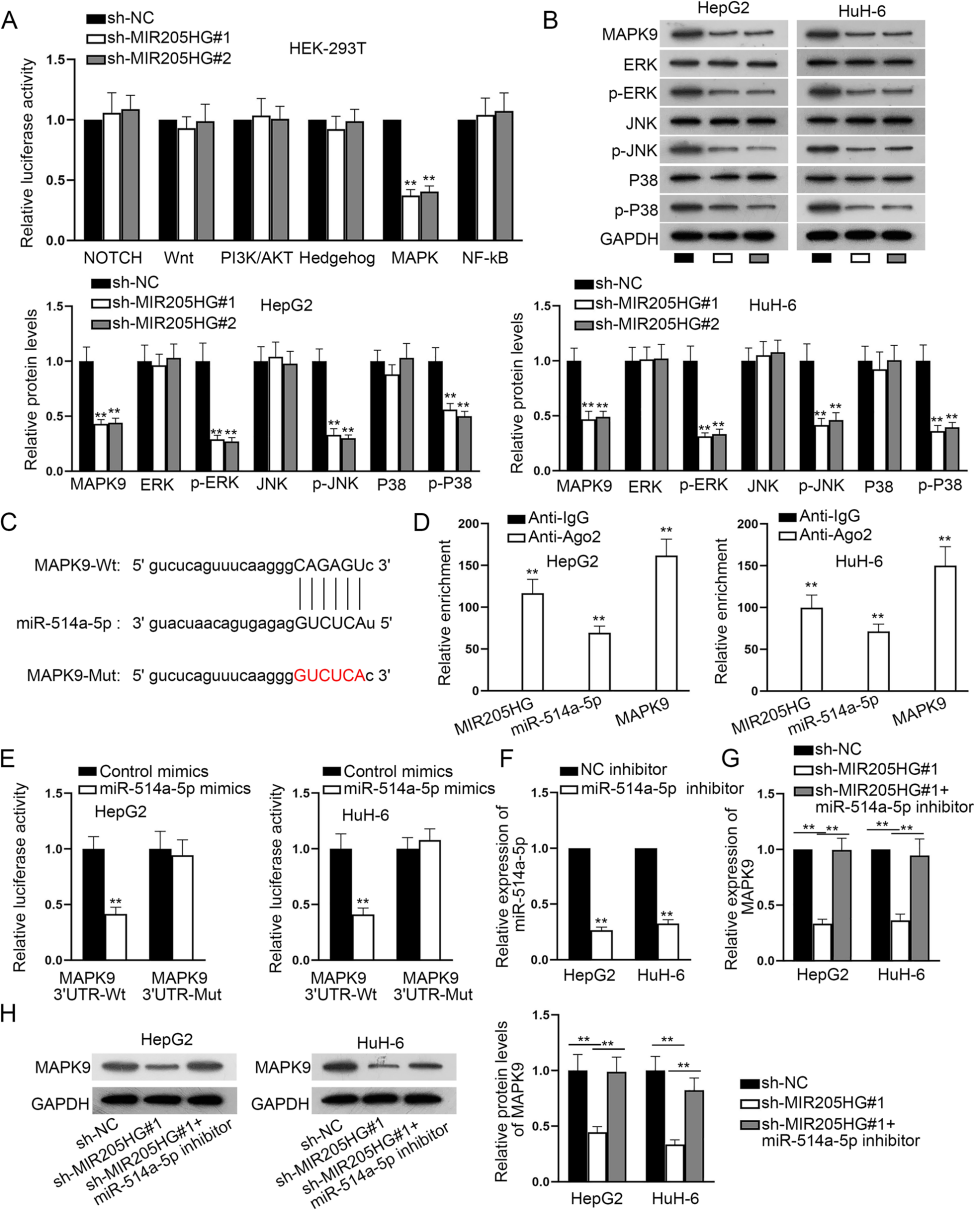
MIR205HG modulates MAPK expression and activates MAPK signaling pathway by competitively binding to miR-514a-5p. **A** The luciferase activities of common signaling pathways were tested when MIR205HG was down-regulated. **B** The protein levels of related factors in the MAPK signaling pathways were analyzed by western blot. The images of original blots were shown in Supplementary File. **C** Binding sites between miR-514a-5p and MAPK9. **D** RIP assay exhibited the relationship among MIR205HG, miR-514a-5p and MAPK9. **E** The interaction between miR-514a-5p and MAPK9 was confirmed by luciferase reporter assay. **F** Transfection of miR-514a-5p inhibitor to lessen miR-514a-5p expression. **G**–**H** MAPK9 expression was detected in HB cells in different groups. GAPDH and U6 were used as internal references for RT-qPCR. Each experiment was performed in triplicate. Student’s t-test, one-way ANOVA and two-way ANOVA were adopted for statistics. **P < 0.01

We conducted luciferase reporter assay for detecting the luciferase activities of common signaling pathways when MIR205HG was depleted. It was discovered that MIR205HG was associated with MAPK signaling pathway for that MIR205HG knockdown led to the decrease in the luciferase activity of MAPK signaling pathway (Fig. 4A). As MAPK9 is a key member of the MAPK signaling pathway, we speculated that MIR205HG might activate MAPK signaling pathway via targeting MAPK9. Western blot showed that after MIR205HG was silenced, the protein levels of MAPK9 and its downstream proteins, p-ERK, p-JNK and p-P38 were all decreased, indicating the positive correlation (Fig. 4B). As depicted in Fig. 4C, the binding regions between miR-514a-5p and MAPK9 were obtained. The results of RIP assay indicated the coexistence of MIR205HG, miR-514a-5p and MAPK9 in RISC, suggesting the ceRNA mode (Fig. 4D). Meanwhile, we performed luciferase reporter assay for the detection of the interaction between miR-514a-5p and MAPK9. The results unearthed that when miR-514a-5p was enhanced, the luciferase activity of MAPK9 3’UTR-Wt was attenuated (Fig. 4E). Then, miR-514a-5p inhibitor was transfected into HB cells to lessen miR-514a-5p expression (Fig. 4F). Subsequently, rescue experiments were performed. RT-qPCR and western blot were applied to detect MAPK9 expression after the transfection of sh-NC, sh-MIR205HG#1 or sh-MIR205HG#1 + miR-514a-5p inhibitor. The data manifested that MIR205HG deficiency led to a decrease on MAPK9 mRNA and protein levels and this effect was completely restored by miR-514a-5p inhibitor (Fig. 4G-H). Taken together, MIR205HG mediates MAPK9 expression by competitively binding to miR-514a-5p.

### MiR-514a-5p down-regulation or MAPK9 up-regulation partially restores the suppressive role of MIR205HG insufficiency in HB progression

**Fig. 5:**
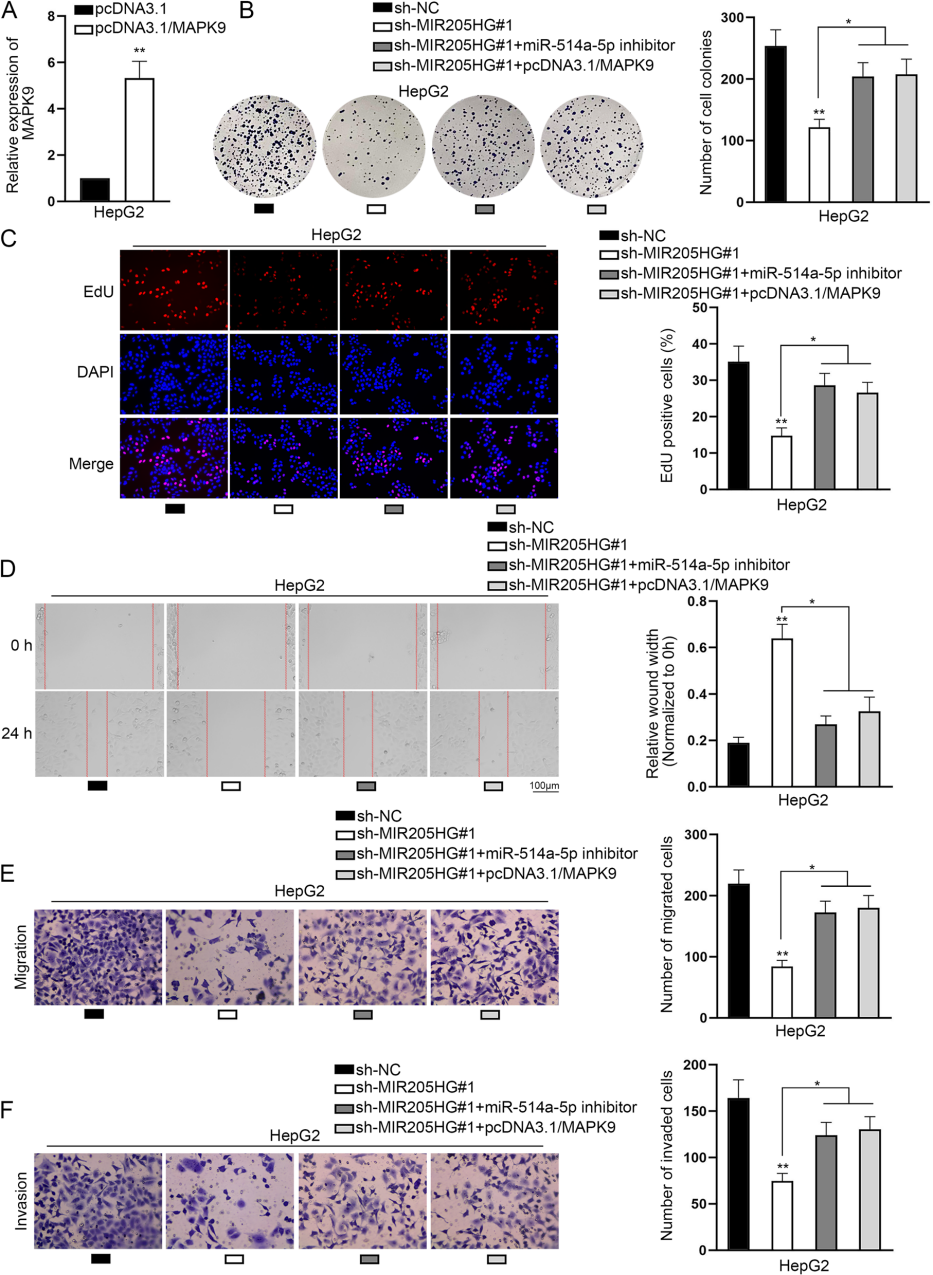
MiR-514a-5p down-regulation or MAPK9 up-regulation partially restores the suppressive role of MIR205HG insufficiency in HB progression. **A** MAPK9 was overexpressed in HepG2 cells. **B**–**C** Cell proliferation was observed in HepG2 cells in the sh-NC group, sh-MIR205HG#1 group, sh-MIR205HG#1 + miR-514a-5p inhibitor group and sh-MIR205HG#1 + pcDNA3.1/MAPK9 group. **D** Wound healing assay assessed cell migration in the sh-NC group, sh-MIR205HG#1 group, sh-MIR205HG#1 + miR-514a-5p inhibitor group and sh-MIR205HG#1 + pcDNA3.1/MAPK9 group. **E**–**F** Cell migration and invasion were appraised in different groups through transwell assays. GAPDH and U6 were used as internal references for RT-qPCR. Each experiment was performed in triplicate. Student’s t-test and one-way ANOVA were adopted for statistics. *P < 0.05, **P < 0.01

To confirm the MIR205HG/miR-514a-5p/MAPK9 axis in HB, rescue assays were carried out. First of all, MAPK9 expression was enhanced in HepG2 cells using pcDNA3.1/MAPK9 (Fig. 5A). Afterwards, we performed functional experiments to detect HB cell progression after the transfection of sh-NC, sh-MIR205HG#1, sh-MIR205HG#1 + miR-514a-5p inhibitor or sh-MIR205HG#1 + pcDNA3.1/MAPK9. As attested by colony formation and EdU assays, MIR205HG depletion suppressed the proliferation of HepG2 cells while this effect was partially countervailed by miR-514a-5p inhibitor or MAPK9 overexpression (Fig. 5B–C). Similarly, wound healing and transwell assays proved that the weakened migratory and invasive capacities of HB cells due to MIR205HG interference were also partially rescued by miR-514a-5p down-regulation or MAPK9 up-regulation (Fig. 5D–F). Overall, MIR205HG contributes to HB development via modulating miR-514a-5p/MAPK9 axis.

### MIR205HG sponges miR-205-5p to activate PI3K/AKT signaling pathway

**Fig. 6:**
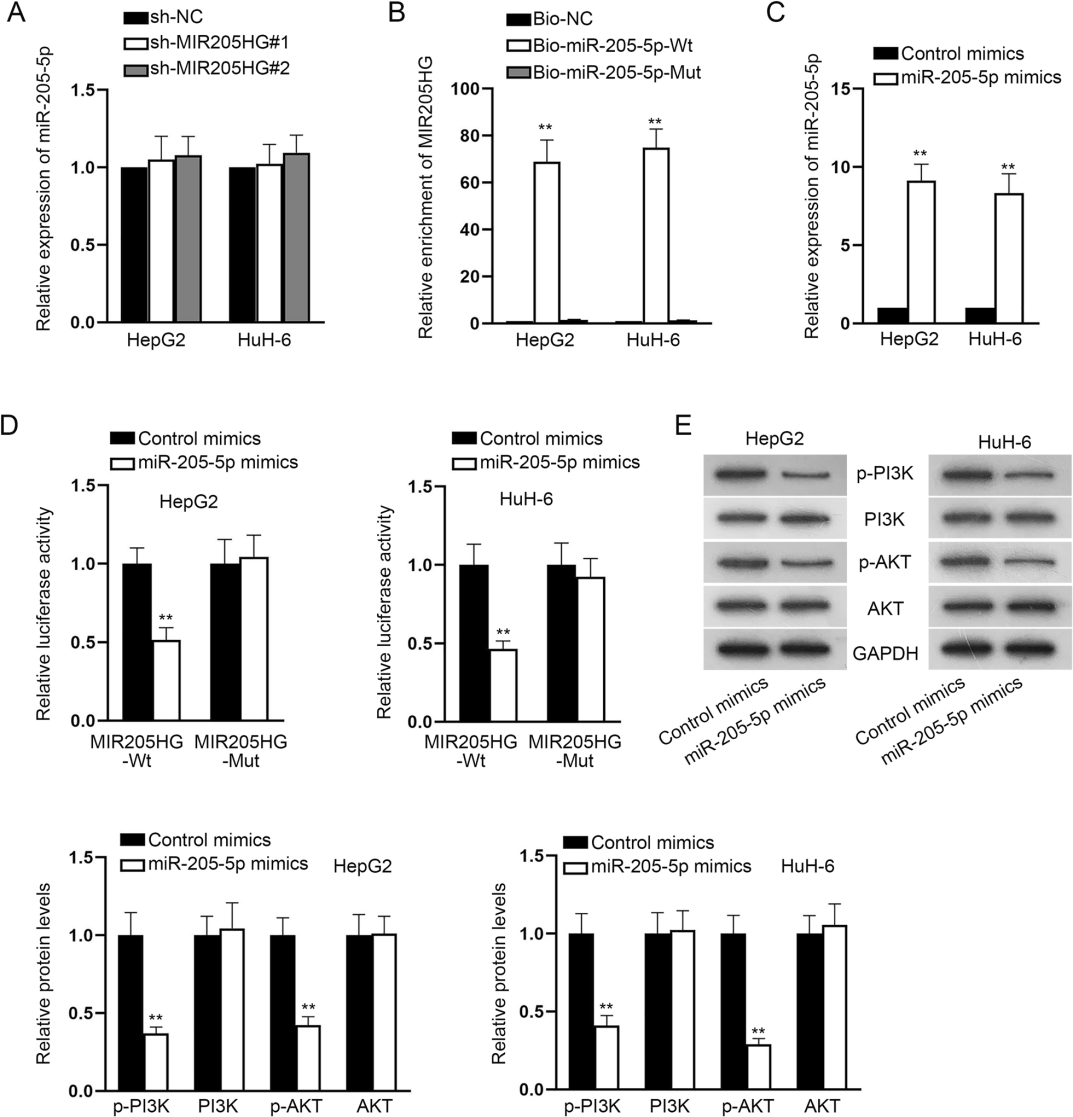
MIR205HG sponges miR-205-5p to activate PI3K/AKT signaling pathway. **A** MiR-205-5p expression was examined after MIR205HG was knocked down. **B** RNA pull down assay detected the accumulation of MIR205HG in the biotin-labeled miR-205-5p-Wt probe and biotin-labeled miR-205-5p-Mut probe. **C** Transfection of miR-205-5p mimics to overexpress miR-205-5p. **D** The luciferase activities in the MIR205HG-Wt group and MIR205HG-Mut group were detected when miR-205-5p was overexpressed. **E** The protein levels of related factors in the PI3K/AKT signaling pathway were analyzed by western blot when miR-205-5p was up-regulated. GAPDH and U6 were used as internal references for RT-qPCR. Each experiment was performed in triplicate. Student’s *t* test, one-way ANOVA and two-way ANOVA were adopted for statistics. **P < 0.01

According to the previous findings, we made a conjecture that miR-205-5p was also regulated by MIR205HG as MIR205HG is a host gene for miR-205. Firstly, we performed RT-qPCR analysis to detect miR-205-5p after the knockdown of MIR205HG. The results suggested that silencing of MIR205HG exerted no influence on miR-205-5p expression (Fig. 6A). We then performed RNA pull down assay to detect the interaction between MIR205HG and miR-205-5p. The results showed that MIR205HG was enriched in biotin-labeled miR-205-5p-Wt probe instead of biotin-labeled miR-205-5p-Mut probe, suggesting the interaction (Fig. 6B). Besides, after the overexpression of miR-205-5p by miR-205-5p mimics, luciferase reporter assay further confirmed the interaction between miR-205-5p and MIR205HG (Fig. 6C–D). It has been reported that MiR-205-5p is involved in PI3K/AKT signaling pathway [18]. We performed western blot analysis to detect the activation level of p-PI3K and p-AKT in HepG2 and HuH-6 cells. The transfection of miR-205-5p mimics inactivated the PI3K/AKT signaling pathway as evidenced by the reduction of p-PI3K and p-AKT (Fig. 6E). To affirm that whether MIR205HG contributed to HB progression by sponging miR-514a-5p and miR-205-5p, rescue assays were implemented. MiR-205-5p expression was firstly reduced after transfection of miR-205-5p inhibitor (Additional file 3: Fig. S2A). Next, we performed functional experiments to detect HB cell progression after the transfection of sh-NC, sh-MIR205HG#1, sh-MIR205HG#1 + miR-205-5p inhibitor or sh-MIR205HG#1 + miR-514a-5p inhibitor + miR-205-5p inhibitor into HepG2 cells. Via performing colony formation and EdU assays, we noticed that down-regulation of miR-205-5p and miR-514a-5p completely offset the inhibited cell proliferation caused by MIR205HG knockdown (Additional file 3: Fig. S2B–C). Likewise, the decrease on cell migration and invasion on account of MIR205HG interference was also countervailed by miR-205-5p inhibitor and miR-514a-5p inhibitor as evidenced by the results of wound healing and transwell assays (Supplementary Fig. 2D-F). Collectively, MIR205HG sponges miR-205-5p.

## Discussion

It is implicated that MIR205HG widely participates in diverse types of cancers. For example, Li et al. have certified that MIR205HG aggravates the process of esophageal cell carcinoma through sponging miR-214 and regulating SOX4 expression (14). Liu et al. have identified that MIR205HG acts as an oncogene in lung squamous cell carcinoma [15]. Dong et al. have proved that MIR205HG influences the biological activities of cervical cancer cells by targeting SRSF1/KRT17 axis [16]. In accordance with these findings, MIR205HG was discovered to be prominently up-regulated in HB cells. Functionally, silencing of MIR205HG impeded cell proliferation, migration and invasion in HB.

Competing endogenous RNA (ceRNA) network is determined as an important regulatory mechanism in cancers [19]. In our study, we observed that MIR205HG was mainly accumulated in the cytoplasm of HB cells. As a result, we speculated that MIR205HG might modulate mRNAs to exert its functions by modulating miRNAs. As expected, miR-514a-5p was selected due to the fact that it was most down-regulated in HB cells among the predicted miRNAs from starBase. The strong affinity of MIR205HG with miR-514a-5p was also confirmed by mechanism assays.

It has been found that MAPK signaling pathway is involved in cell malignant phenotypes, including cell proliferation, cell differentiation and cell migration [20]. As a key member in the MAPK signaling pathway, MAPK9 is also correlated with several diseases, including non-small-cell lung carcinoma [21], haemophilia A [22], and type I diabetes [23]. Through our investigation, MAPK9 was discovered to be a downstream target of miR-514a-5p. Thence, MIR205HG activates MAPK signaling pathway via targeting miR-514a-5p and regulating MAPK9 expression. Besides, down-regulation of miR-514a-5p or up-regulation of MAPK9 partially restored the suppressed cell proliferation, migration and invasion on account of MIR205HG depletion.

It is acknowledged that MIR205HG acts as the host gene of miR-205. Meanwhile, miR-205-5p has been reported to play suppressive roles in many cancers. For instance, miR-205-5p suppresses the development of prostatic carcinoma cells via being regulated by p63 or targeting ZEB1 [24] [25]; moreover, the cell progression of osteosarcoma [26] and pancreatic cancer [27] have been reported to be inhibited by miR-205-5p. Similarly, we demonstrated that miR-205-5p also interacted with MIR205HG and participated in the activation of PI3K/AKT signaling pathway. Eventually, rescue assays attested that ablation of miR-514a-5p and miR-205-5p completely counteracted the suppressive role of MIR205HG insufficiency in HB progression.

## Conclusion

All in all, MIR205HG was highly expressed in HB cells. Functionally, MIR205HG interference hampered the proliferative, migratory and invasive capacities of HB cells. From the perspective of mechanism, MIR205HG competitively bound to miR-514a-5p to modulate MAPK9 expression and stimulate MAPK signaling pathway. Besides, MIR205HG had a binding with miR-205-5p and activated PI3K/AKT signaling pathway.

## Methods

### Cell lines and culture

HB cell lines including HepG2, HuH-7, HuH-1, HuH-6 and normal epithelial cell line (THLE-3) were selected for this investigation. HepG2 and THLE-3 cell lines were both obtained from ATCC (Manassas, VA, USA) while HuH-7, HuH-1, HuH-6 cell lines were purchased from Huatuo Biological Technology Co., Ltd. (Shenzhen, China). Among them, HepG2 and THLE-3 cell lines were cultured in Eagle’s Minimum Essential Medium (EMEM) and BEGM respectively. HuH-7 and HuH-6 cells were incubated in Dulbecco’s Modified Eagle’s medium (DMEM) while HuH-1 cell line was cultured in RPMI-1640 Medium. All mediums contained 10% fetal bovine serum (FBS) and incubated at 37 °C with 5% CO_2_.

### Transfection of cells

The knockdown of MIR205HG in HB cells was implemented by transfection with sh-MIR205HG#1/2/3 plasmids acquired from Ribobio (Guangzhou, China). MiR-514a-5p mimics/inhibitor, miR-205-5p mimics/inhibitor and their respective negative controls were commercially obtained from GenePharma (Shanghai, China). Furthermore, pcDNA3.1 vectors were obtained from Invitrogen to overexpress MAPK9. Lipofectamine 2000 was used to transfect HB cells as per the instruction of manufacturer.

### RNA extraction and quantitative real-time PCR (RT-qPCR)

The total RNA was subjected to extraction from cells using Trizol reagent. PrimeScript™ RT Master Mix (TaKaRa, Shiga, Japan) was used to obtain cDNA through reverse transcribing two mircrograms of total RNA. SYBR Premix Ex TaqTM II (Takara Bio, Tokyo, Japan) was applied to perform RT-qPCR. GAPDH and U6 were utilized as internal references. The 2^−ΔΔCt^ method was used to evaluate gene expression. The experiment was implemented in triplicate.

### Colony formation assay

In brief, cultured cells were inoculated into 6-well plates. After 14 days of incubation, cells were subjected to fixation using ethanol, followed by the staining with crystal violet. Finally, the stained cell colonies were observed and counted. The experiment was independently conducted in triplicate.

### 5-ethynyl-20-deoxyuridine (EdU) staining assay

Cells at logarithmic growth stage were taken and seeded in 96-well plates with 4 × 10^3^ ~ 1 × 10^5^ cells per well. Subsequently, the cells were cultured to the normal growth stage. A total of 50 μM EdU culture medium was prepared via the dilution of EdU solution (reagent A) by 1:1000. The cells were subjected to fixation, followed by the staining using Apollo. The assay was independently conducted in triplicate.

### Wound healing assay

A total of 3 × 10^3^ cells were seeded into 6-well plates and cultured in serum-free medium for 24 h at 37 °C. When cells reached 80% confluence, a straight scratch wound was made. Then cells were cultured for another 24 h, and scratches were monitored and photographed at 0 and 24 h. An inverted microscope (DMi1, Leica, Wetzlar, Germany) at 100 × magnification was used to monitor the wound healing. The assay was independently conducted in triplicate.

### Transwell assay

The transfected HB cells were harvested and put into the upper chamber of Transwell inserts. Afterwards, 10% FBS was added to the lower chamber. The chambers coated with Matrigel (BD Biosciences, San Diego, CA, USA) were used for invasion assay, while without Matrigel for migration assay. After 24 h, migrated or invaded cells were finally visualized using optical microscope (Olympus) after being stained by crystal violet. The assay was independently conducted in triplicate.

### Subcellular fractionation

Cytoplasmic and nuclear RNA were isolated with the employment of PARIS™ Kit (Ambion, Austin, TX, USA) in line with the manufacturer’s protocols. Cells were washed in PBS. Afterwards, cell samples were treated with cell fractionation buffer and disruption buffer. GAPDH and U6 were seen as the cytoplasmic control and the nuclear control respectively. The isolated RNA in the nucleus and the cytoplasm was measured by PCR analysis. The assay was independently conducted in triplicate.

### Fluorescent in situ hybridization (FISH)

RNA FISH probe (Ribobio) targeting MIR205HG was designed and utilized as per the guide of provider. After hybridization using FISH probe, cells were counterstained by DAPI solution, followed by the detection using fluorescence microscope (Olympus). The experiment was independently performed in triplicate.

### RNA pull down assay

RNA pull down assay was implemented by use of Pierce Magnetic RNA–Protein Pull-Down Kit (Thermo Fisher Scientific, Waltham, MA, USA). The cell extracts from HepG2 and HuH-6 cells were used for incubation with biotinylated probes of miR-514a-5p-WT/Mut or miR-205-5p-WT/Mut. After adding magnetic beads, relative RNA enrichment was assessed by RT-qPCR. The experiment was independently performed in triplicate.

### RNA-binding protein immunoprecipitation (RIP)

Z-Magna RIP™ RNA-binding Protein Immunoprecipitation kit (Millipore Corporation, USA) was adopted to perform RIP assay. Anti-Ago2 (Abcam) antibody as well as anti-IgG (Abcam) antibody were used for immunoprecipitation with cell lysates. Finally, the RNA complexes were subjected to extraction for RT-qPCR analysis. The experimental procedure was independently carried out in triplicate.

### Luciferase reporter assay

MIR205HG or MAPK9 sequence with the wild-type (Wt) and mutant type (Mut) miR-514a-5p binding sites was inserted into pmirGLO dual-luciferase vector to form pmirGLO-MIR205HG Wt/Mut and pmirGLO-MAPK9 3’-UTR-Wt/Mut respectively. Later, miR-514a-5p mimics or control mimics were co-transfected with the reporter construct into HB cells. Similarly, MIR205HG sequence of Wt and Mut of miR-205-5p was also utilized to pmirGLO-MIR205HG Wt/Mut and then was co-transfected with NC mimics and miR-205-5p mimics into HB cells. After 48 h, luciferase activity was measured via the employment of the Dual Luciferase Reporter Assay Kit (Promega, Madison, WI, USA). The assay was independently conducted in triplicate.

### Western blot analysis

Separated protein samples were subjected to transference to PVDF membranes (Millipore, Bedford, MA, USA). After being blocked with skimmed milk, the membranes were subjected to incubation with the following primary antibodies against MAPK9, ERK, p-ERK, JNK, p-JNK, P38, p-P38, p-PI3K, PI3K, p-AKT, AKT and GAPDH. Subsequently, the blots were subjected to incubation with secondary antibody. At last, Chemiluminescence system (GE Healthcare, Chicago, USA) was applied for the quantification of proteins. The assay was independently conducted in triplicate.

### In vivo study

Ten male BALB/c nude mice (4–5 weeks old) were purchased from the Beijing Chrles River Experimental Animal Technology Co., LTD. A total of 1 × 10^7^ HepG2 cells stably transfected with sh-NC or sh-MIR205HG#1 were independently injected subcutaneously into the right back of each nude mouse. Seven days after the injection, tumor volume was recorded every 3 days. Twenty-eight days after the injection, all the mice were sacrificed for tumor weighing.

### Statistical analysis

Experimental data were subjected to analysis by use of SPSS 22.0 statistical software package. All data were presented as mean ± standard deviation (SD). For differences comparison between two groups or more, Student’s t-test or ANOVA was adopted. All the experiments were independently implemented in triplicate. Differences were considered to be statistically significant when *P* < 0.05.

## Supplementary Information

**Additional file 1**. Untrimmed whole western blots for Figure 4B.

**Additional file 2**. **Figure S1** Gain-of-function experiments and in vivo assays for the evaluation of the role of MIR205HG in HB. (A) MIR205HG expression was up-regulated by pcDNA3.1/MIR205HG in THLE-3 cells. (B) EdU assays evaluated the proliferative capacity of THLE-3 cells after MIR205HG was overexpressed. (C-D) Transwell assays were implemented to observe the migration and invasion of THLE-3 cells after the overexpression of MIR205HG. (E) The tumor growth in sh-NC and sh-MIR205HG#1 groups was recorded. (F) The tumor weight was measured in sh-NC and sh-MIR205HG#1 groups. GAPDH and U6 were used as internal references for RT-qPCR. Each experiment was performed in triplicate. Student’s t-test was adopted for statistics. **P < 0.01.

**Additional file 3**. **Figure S2** Down-regulation of miR-514a-5p and miR-205-5p completely rescues the influence of MIR205HG deficiency on HB progression. (A) Transfection of miR-205-5p inhibitor reduces miR-205-5p expression. (B-C) Cell proliferation was evaluated in HepG2 cells in the sh-NC group, sh-MIR205HG#1 group, sh-MIR205HG#1 + miR-205-5p inhibitor group and sh-MIR205HG#1 + miR-205-5p inhibitor + miR-514a-5p inhibitor group. (D) Wound healing assay detected cell migration in the sh-NC group, sh-MIR205HG#1 group, sh-MIR205HG#1 + miR-205-5p inhibitor group and sh-MIR205HG#1 + miR-205-5p inhibitor + miR-514a-5p inhibitor group in HepG2 cells. (E–F) The migratory and invasive capacities of HB cells were appraised in different groups. GAPDH and U6 were used as internal references for RT-qPCR. Each experiment was performed in triplicate. Student’s t-test and one-way ANOVA were adopted for statistics. *P < 0.05, **P < 0.01.

## Data Availability

Not applicable.

## Abbreviations

HB: Hepatoblastoma
lncRNAs: Long non-coding RNAs
MIR205HG: MIR205 host gene
miR-514a-5p: MicroRNA-514a-5p
MAPK9: Mitogen-activated protein kinase 9
miR-205-5p: MicroRNA-205-5p
ceRNA: Competing endogenous RNA
ATCC: American Type Culture Collection
EMEM: Eagle’s Minimum Essential Medium
DMEM: Dulbecco’s Modified Eagle’s medium
FBS: Fatal Bovine Serum
RT-qPCR: Quantitative real-time PCR
EdU: 5-Ethynyl-20-deoxyuridine
FISH: Fluorescence in situ hybridization
RIP: RNA immunoprecipitation
Wt: Wild-type
Mut: Mutant type
SD: Standard deviation

## References

[1] Sharma D, Subbarao G, Saxena R (2017). Hepatoblastoma. Semin Diagn Pathol.

[2] Stocker JT (1994). Hepatoblastoma. Semin Diagn Pathol.

[3] Herzog CE, Andrassy RJ, Eftekhari F (2000). Childhood cancers: hepatoblastoma. Oncologist.

[4] Abbasoğlu L, Gün F, Tansu Salman F, Relik A, Saraq F, Unüvar A (2004). Hepatoblastoma in children. Acta Chir Belg.

[5] von Schweinitz D (2012). Hepatoblastoma: recent developments in research and treatment. Semin Pediatr Surg.

[6] Geiger JD (1996). Surgery for hepatoblastoma in children. Curr Opin Pediatr.

[7] Choudhari R, Sedano MJ, Harrison AL, Subramani R, Lin KY, Ramos EI (2020). Long noncoding RNAs in cancer: From discovery to therapeutic targets. Adv Clin Chem.

[8] Li T, Chen Y, Zhang J, Liu S (2018). LncRNA TUG1 promotes cells proliferation and inhibits cells apoptosis through regulating AURKA in epithelial ovarian cancer cells. Medicine.

[9] Luo Y, Wang Q, Teng L, Zhang J, Song J, Bo W (2020). LncRNA DANCR promotes proliferation and metastasis in pancreatic cancer by regulating miRNA-33b. FEBS Open Bio.

[10] Yan S, Shan X, Chen K, Liu Y, Yu G, Chen Q (2018). LINC00052/miR-101-3p axis inhibits cell proliferation and metastasis by targeting SOX9 in hepatocellular carcinoma. Gene.

[11] Zhang Z, Liu F, Yang F, Liu Y (2018). Kockdown of OIP5-AS1 expression inhibits proliferation, metastasis and EMT progress in hepatoblastoma cells through up-regulating miR-186a-5p and down-regulating ZEB1. Biomed Pharmacother.

[12] Chen LJ, Yuan MX, Ji CY, Zhang YB, Peng YM, Zhang T (2020). Long Non-Coding RNA CRNDE Regulates Angiogenesis in Hepatoblastoma by Targeting the MiR-203/VEGFA Axis. Pathobiol: J Immunopathol Mol Cell Biol.

[13] Cui X, Wang Z, Liu L, Liu X, Zhang D, Li J (2019). The Long Non-coding RNA ZFAS1 Sponges miR-193a-3p to Modulate Hepatoblastoma Growth by Targeting RALY via HGF/c-Met Pathway. Front Cell Develop Biol.

[14] Li H, Jia J, Yang L, Chu J, Sheng J, Wang C (2020). LncRNA MIR205HG Drives Esophageal Squamous Cell Carcinoma Progression by Regulating miR-214/SOX4 Axis. Onco Targets Ther.

[15] Liu L, Li Y, Zhang R, Li C, Xiong J, Wei Y (2020). MIR205HG acts as a ceRNA to expedite cell proliferation and progression in lung squamous cell carcinoma via targeting miR-299-3p/MAP3K2 axis. BMC Pulm Med.

[16] Dong M, Dong Z, Zhu X, Zhang Y, Song L (2019). Long non-coding RNA MIR205HG regulates KRT17 and tumor processes in cervical cancer via interaction with SRSF1. Exp Mol Pathol.

[17] Michlewski G, Cáceres JF (2019). Post-transcriptional control of miRNA biogenesis. RNA (New York, NY).

[18] Huang J, Wang X, Wen G, Ren Y (2019). miRNA-205-5p functions as a tumor suppressor by negatively regulating VEGFA and PI3K/Akt/mTOR signaling in renal carcinoma cells. Oncol Rep.

[19] Ergun S, Oztuzcu S (2015). Oncocers: ceRNA-mediated cross-talk by sponging miRNAs in oncogenic pathways. Tumour Biol.

[20] Sun Y, Liu WZ, Liu T, Feng X, Yang N, Zhou HF (2015). Signaling pathway of MAPK/ERK in cell proliferation, differentiation, migration, senescence and apoptosis. J Recept Signal Transduct Res.

[21] Wang ZX, Zhao Y, Wang YB, Zhang Q, Zou QX, Liang FH (2020). CircRNF20 aggravates the progression of non-small-cell lung carcinoma by activating MAPK9. Eur Rev Med Pharmacol Sci.

[22] Zhao M, Zhang Y, Liu Y, Sun G, Tian H, Hong L (2019). Polymorphisms in MAPK9 (rs4147385) and CSF1R (rs17725712) are associated with the development of inhibitors in patients with haemophilia A in North China. Int J Lab Hematol.

[23] Jaeschke A, Rincón M, Doran B, Reilly J, Neuberg D, Greiner DL (2005). Disruption of the Jnk2 (Mapk9) gene reduces destructive insulitis and diabetes in a mouse model of type I diabetes. Proc Natl Acad Sci USA.

[24] Liu M, Jia J, Wang X, Liu Y, Wang C. Long non-coding RNA HOTAIR promotes cervical cancer progression through regulating BCL2 via targeting miR-143–3p. Cancer Biol Therapy. 2018. 10.1080/15384047.2018.1423921 PMC5915047 29336659

[25] Tucci P, Agostini M, Grespi F, Markert EK, Terrinoni A, Vousden KH (2012). Loss of p63 and its microRNA-205 target results in enhanced cell migration and metastasis in prostate cancer. Proc Natl Acad Sci USA.

[26] Liu YP, Wan J, Long F, Tian J, Zhang C (2020). circPVT1 Facilitates Invasion and Metastasis by Regulating miR-205-5p/c-FLIP Axis in Osteosarcoma. Cancer Manag Res.

[27] Zhu H, Shan Y, Ge K, Lu J, Kong W, Jia C. LncRNA CYTOR promotes pancreatic cancer cell proliferation and migration by sponging miR-205–5p. Pancreatol: Off J Int Assoc Pancreatol (IAP) 2020;20(6):1139–48. 10.1016/j.pan.2020.05.004 32732173

