# Supplementary figures and images for: LncRNA MIR205HG accelerates cell proliferation, migration and invasion in hepatoblastoma through the activation of MAPK signaling pathway and PI3K/AKT signaling pathway
Source: Biol Direct. 2022 Jan 7;17:2. doi: 10.1186/s13062-021-00309-3 (PMC8740508; doi:10.1186/s13062-021-00309-3)

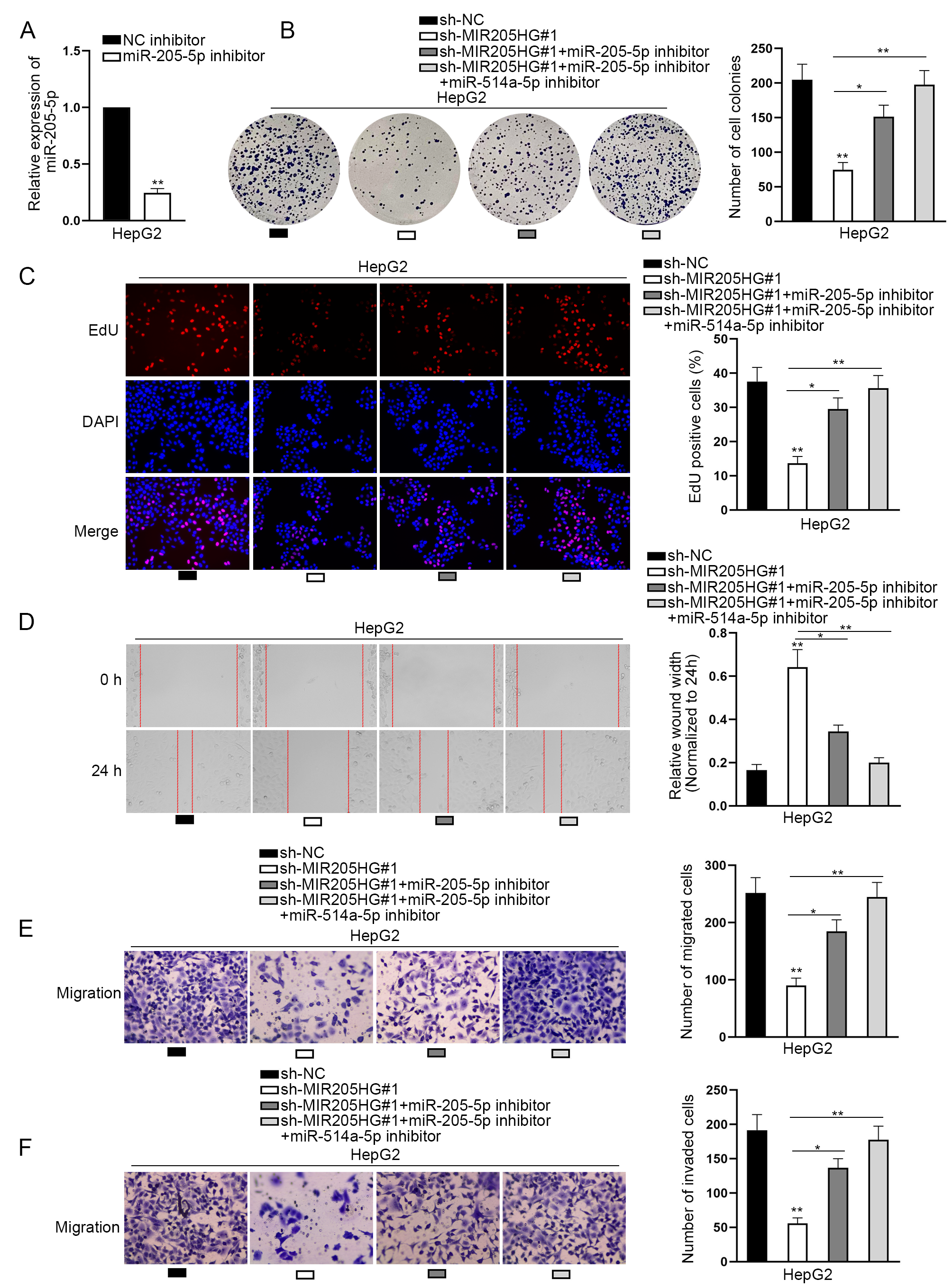

Supplement: Supplementary file 2 — Additional file 2. Figure S1 Gain-of-function experiments and in vivo assays for the evaluation of the role of MIR205HG in HB. (A) MIR205HG expression was up-regulated by pcDNA3.1/MIR205HG in THLE-3 cells. (B) EdU assays evaluated the proliferative capacity of THLE-3 cells after MIR205HG was overexpressed. (C-D) Transwell assays were implemented to observe the migration and invasion of THLE-3 cells after the overexpression of MIR205HG. (E) The tumor growth in sh-NC and sh-MIR205HG#1 groups was recorded. (F) The tumor weight was measured in sh-NC and sh-MIR205HG#1 groups. GAPDH and U6 were used as internal references for RT-qPCR. Each experiment was performed in triplicate. Student’s t-test was adopted for statistics. **P < 0.01. [file 13062_2021_309_MOESM2_ESM.tif]

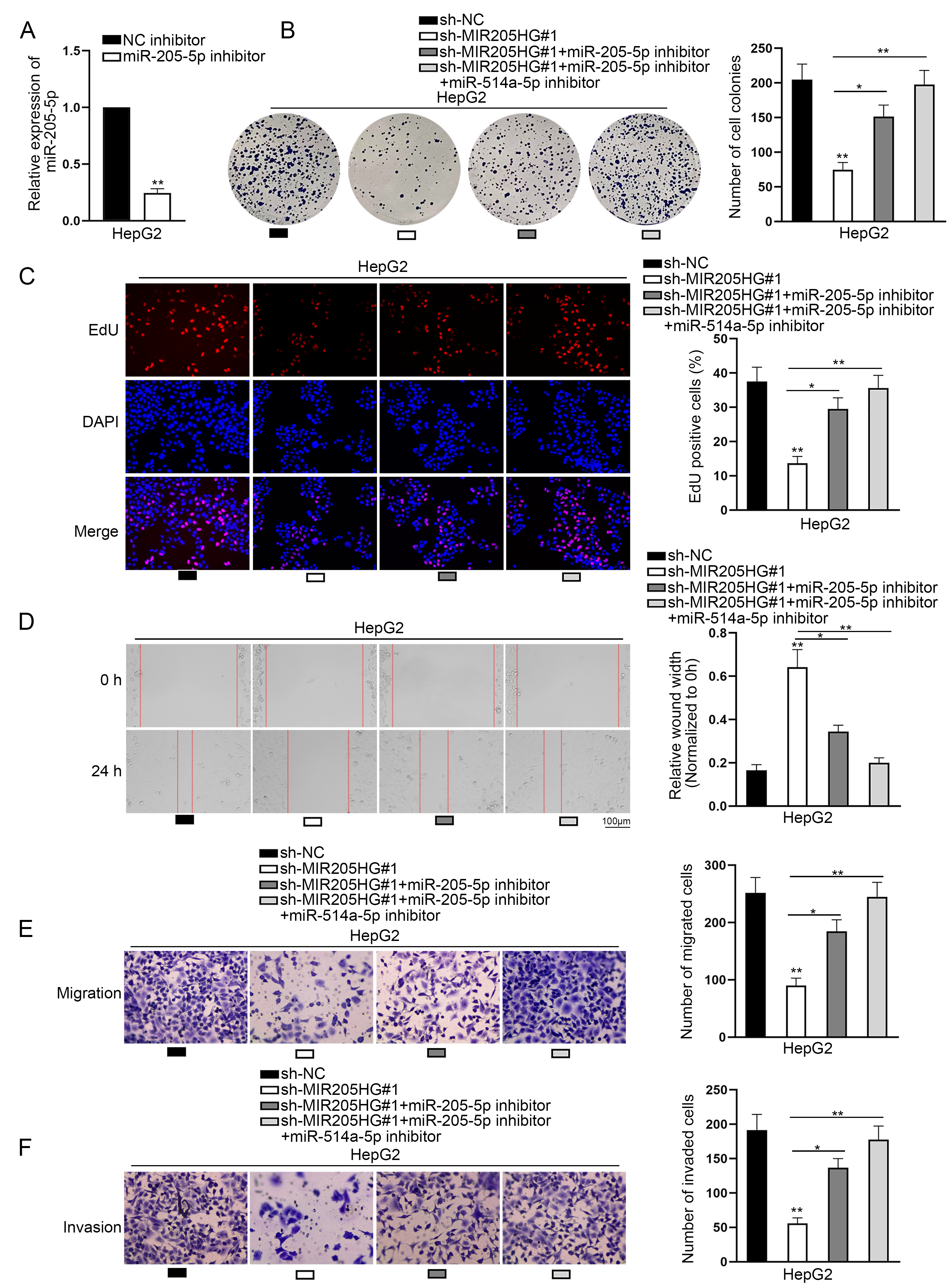

Supplement: Supplementary file 3 — Additional file 3. Figure S2 Down-regulation of miR-514a-5p and miR-205-5p completely rescues the influence of MIR205HG deficiency on HB progression. (A) Transfection of miR-205-5p inhibitor reduces miR-205-5p expression. (B-C) Cell proliferation was evaluated in HepG2 cells in the sh-NC group, sh-MIR205HG#1 group, sh-MIR205HG#1 + miR-205-5p inhibitor group and sh-MIR205HG#1 + miR-205-5p inhibitor + miR-514a-5p inhibitor group. (D) Wound healing assay detected cell migration in the sh-NC group, sh-MIR205HG#1 group, sh-MIR205HG#1 + miR-205-5p inhibitor group and sh-MIR205HG#1 + miR-205-5p inhibitor + miR-514a-5p inhibitor group in HepG2 cells. (E–F) The migratory and invasive capacities of HB cells were appraised in different groups. GAPDH and U6 were used as internal references for RT-qPCR. Each experiment was performed in triplicate. Student’s t-test and one-way ANOVA were adopted for statistics. *P < 0.05, **P < 0.01. [file 13062_2021_309_MOESM3_ESM.tif]
